# Enhancing tumor photodynamic synergistic therapy efficacy through generation of carbon radicals by Prussian blue nanomedicine

**DOI:** 10.1093/rb/rbae103

**Published:** 2024-08-24

**Authors:** Jun Zhong, Mingzhi Zhu, Jiaqi Guo, Xinyu Chen, Ruimin Long, Fabian Körte, Shibin Wang, Hao Chen, Xin Xiong, Yuangang Liu

**Affiliations:** College of Chemical Engineering, Huaqiao University, Xiamen 361021, China; College of Chemical Engineering, Huaqiao University, Xiamen 361021, China; College of Chemical Engineering, Huaqiao University, Xiamen 361021, China; College of Chemical Engineering, Huaqiao University, Xiamen 361021, China; College of Chemical Engineering, Huaqiao University, Xiamen 361021, China; NMI Natural and Medical Sciences Institute, University of Tübingen, Reutlingen 72770, Germany; College of Materials Science and Engineering, Huaqiao University, Xiamen 361021, China; Institute of Pharmaceutical Engineering, Huaqiao University, Xiamen 361021, China; Fujian Provincial Key Laboratory of Biochemical Technology, Xiamen 361021, China; Fujian Provincial Key Laboratory of Intelligent Identification and Control of Complex Dynamic System, Haixi Institutes, Chinese Academy of Sciences, Quanzhou 362200, China; NMI Natural and Medical Sciences Institute, University of Tübingen, Reutlingen 72770, Germany; College of Chemical Engineering, Huaqiao University, Xiamen 361021, China; Institute of Pharmaceutical Engineering, Huaqiao University, Xiamen 361021, China; Fujian Provincial Key Laboratory of Biochemical Technology, Xiamen 361021, China

**Keywords:** artesunate, Prussian blue, carbon radicals, photodynamic therapy

## Abstract

Significant progress has been achieved in tumor therapies utilizing nano-enzymes which could convert hydrogen peroxide into reactive oxygen species (ROS). However, the ROS generated by these enzymes possess a short half-life and exhibit limited diffusion within cells, making it challenging to inflict substantial damage on major organelles for effective tumor therapy. Therefore, it becomes crucial to develop a novel nanoplatform that could extend radicals half-life. Artesunate (ATS) is a Fe (II)-dependent drug, while the limited availability of iron (II), coupled with the poor aqueous solubility of ATS, limits its application. Here, Prussian blue (PB) was selected as a nano-carrier to release Fe (II), thus constructing a hollow Prussian blue/artesunate/methylene blue (HPB/ATS/MB) nanoplatform. HPB degraded and released iron(III), ATS and MB, under the combined effects of NIR irradiation and the unique tumor microenvironment. Moreover, Fe (III) exploited GSH to formation of Fe (II), disturbing the redox homeostasis of tumor cells and Fe (II) reacted with H_2_O_2_ and ATS to generate carbon radicals with a long half-life *in situ*. Furthermore, MB generates ^1^O_2_ under laser irradiation conditions. *In vitro* and *in vivo* experiments have demonstrated that the HPB/ATS/MB NPs exhibit a synergistic therapeutic effect through photothermal therapy, photodynamic therapy and radical therapy.

## Graphical abstract

**Figure:**
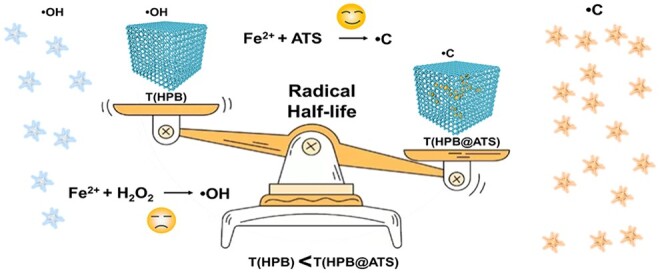

## Introduction

Nowadays, cancer poses a significant challenge in the healthcare system and remains one of the most pressing global health issues [1]. Despite remarkable advancements in cancer treatment over the past decade, chemotherapy remains the predominant approach [2]. While targeted therapy and immunotherapy have emerged as alternative treatments, their high cost and limited applicability hinder their widespread adoption. Targeted therapies, for instance, are only effective against cancers with specific biomarkers, whereas immunotherapy exhibits limited efficacy in most patients [3]. Furthermore, the development of new anti-cancer drugs entails substantial costs and requires extensive research on pharmacology, pharmacokinetics, and safety, along with time-consuming processes from target identification to phase III clinical trials. Consequently, exploring and repurposing well-established drugs that have already been extensively utilized in clinical practice represents an efficient and suitable approach for drug development [4–7].

Artemisinin stands out as a promising candidate for repurposing studies due to its outstanding safety and efficacy in antimalarial treatments [8]. Initially isolated and extracted from Artemisia annua in 1971, artemisinin is a sesquiterpene lactone compound with a unique endoperoxide bridge (-C-O-O-C) structure that confers significant antimalarial efficacy. Recent trends in drug repurposing have unveiled the expansive potential of artemisinin in cancer treatment [9], antibacterial applications [10], antifibrotic effects [11], antiviral properties [12] and anti-inflammatory effects, further expanding its utility in cancer therapy.

Since the initial reports of potential anticancer properties of artemisininoids in 1993, numerous studies have confirmed the potential of artemisinin as a novel cancer treatment [13, 14]. However, the short half-life and limited solubility of artemisinin significantly impact its treatment duration [15]. To overcome these limitations, researchers have modified artemisinin by removing carbonyl groups, resulting in dihydroartemisinin (DHA) [16]. Subsequently, further modifications led to the development of artesunate (ATS), addressing the shortcomings of the original artemisinin compound [17, 18]. Studies have demonstrated the promising activity of ATS against various cancer cell lines [19]. Nevertheless, the specific cytotoxicity of ATS and iron towards cancer cells remains inadequately understood. Multiple pathways contribute to the pharmacotoxicity in cancer cells, including oxidative stress [8], DNA damage, anti-angiogenesis, induction of apoptosis and autophagy, ROS production leading to iron death [20] and cell cycle arrest [21–23]. The endoperoxide group in the structure of ATS is believed to play a crucial role in its pharmacological activity [24]. Activation of ATS drug activity results in the breakage of the characteristic (-C-O-O-C-) group, forming a carbon-centered free radical (a type of reactive oxygen species [ROS]) capable of alkylating proteins in cells and disrupting normal cellular metabolic functions [25]. Importantly, the toxic effect of ATS on cancer cells can be significantly enhanced by iron nanocarrier, leading to more than a 100-fold increase in toxicity with higher iron concentrations. Furthermore, Efferth *et al.* [26] demonstrated that the regulation of intracellular iron levels through iron chelators or exogenous iron can mediate the antitumor effects of artemisinin. These experimental findings highlight the close relationship between the pharmacological effects of ATS and Fe^2+^.

Despite its potential, the further application of ATS in anti-tumor therapy is hindered by its poor water solubility and limited availability of intracellular ferrous ions. To overcome these challenges, effective delivery of ATS to the tumor site and the provision of exogenous iron activation to enhance its cancer cell toxicity have become research priorities. ATS-based nanocomplexes hold promise as successful nano-drug delivery systems for anti-tumor drugs [27]. Prussian blue (PB), an inexpensive and easily synthesized antidote approved by the US Food and Drug Administration (FDA) for clinical treatment of thallium and radioactive cesium poisoning, has gained attention due to its excellent biocompatibility, safety, stability and ease of surface function modification [28–30]. PB exhibits a porous network stereostructure and is composed of iron, ferrous and cyanide ions within a face-centered cubic unit cell, making it an ideal ferrous carrier.

**Scheme 1.**
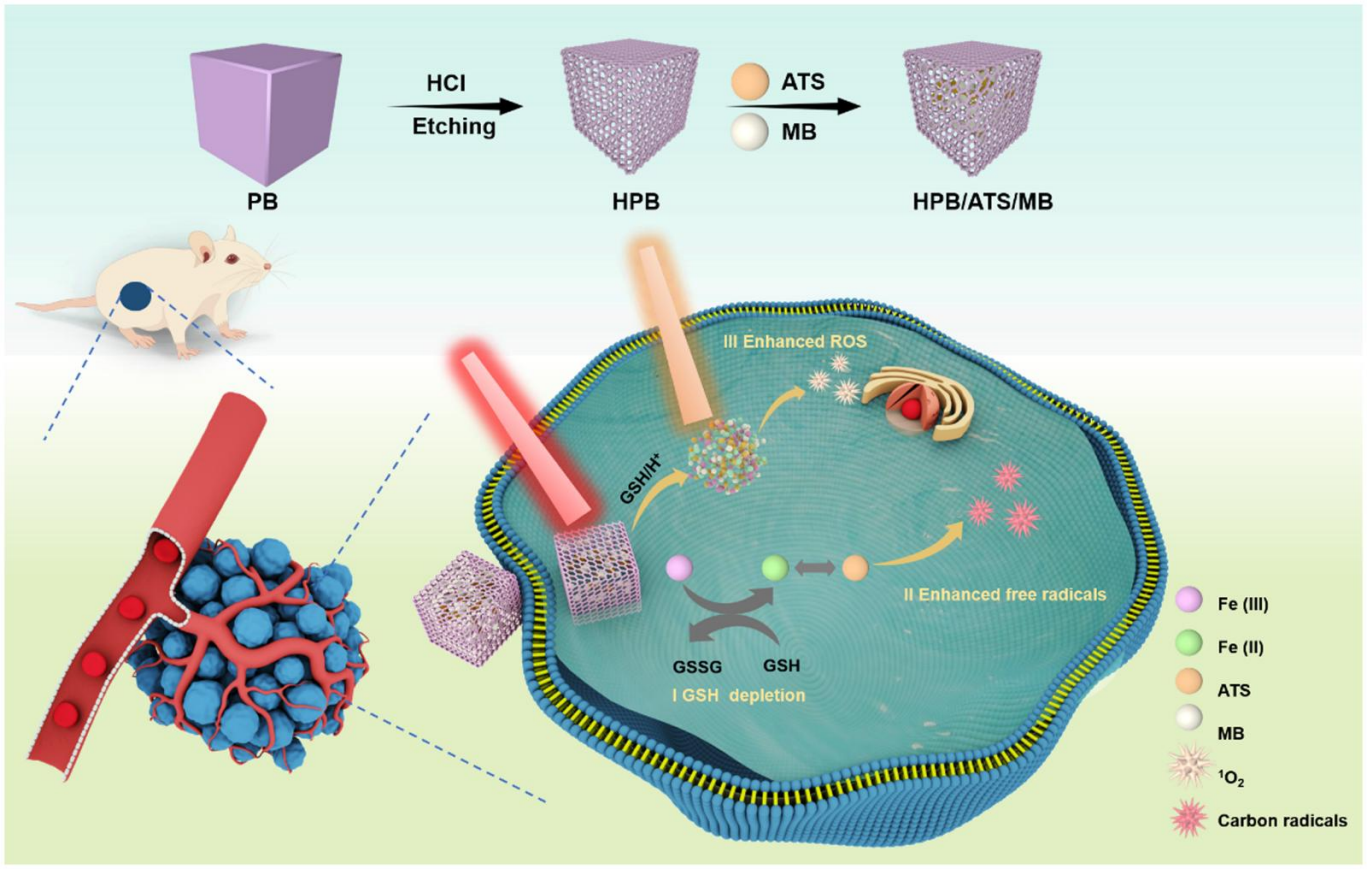
PB nanomedicine for *in situ* carbon radical generation and photodynamic/photothermal antitumor amplification.

Therefore, this study aimed to construct a hollow Prussian blue/artesunate/methylene blue (HPB/ATS/MB) nanocarrier using PB as the carrier to deliver the water-insoluble drug ATS to the tumor site. PB serves as a carrier, a photothermal agent and an exogenous iron agent, converting light energy into heat energy under laser irradiation and inducing cell death through photothermal therapy (PTT) [31]. Within tumor cells, the HPB vector releases Fe^2+^ under the combined action of photothermal effects and the acidic tumor environment, thereby activating and enhancing the selective killing effect of ATS on tumor cells, ultimately inducing apoptosis through the generation of carbon-based free radicals. Additionally, HPB incorporates the photosensitizer MB, which sensitizes oxygen to produce singlet oxygen, exerting toxic effects on cells and enhancing the effectiveness of cancer treatment. *In vitro* and *in vivo* experiments demonstrate the excellent tumor diagnosis and treatment effects of the HPB/ATS/MB nanodrug carrier system, which realizes the synergistic treatment effect combining PTT, photodynamic therapy (PDT) and free radical therapy (Scheme 1).

## Materials and methods

### Materials

Polyvinylpyrrolidone (PVP) and potassium ferricyanide (K_3_[Fe(CN)_6_]) were purchased from Aladdin (Shanghai, China). Hydrochloric acid (HCl, 99.7%), ethanol (99.9%), ATS and methylene blue were obtained from Sinopharm Chemical Reagent Co., Ltd, China. 1,3-diphenylisobenzofuran (DPBF, GR) was bought from Admas (Shanghai, China). Dulbecco’s modified eagle medium (DMEM), trypsin-EDTA solution, fetal bovine serum (FBS), penicillin-streptomycin, 2′,7′-dichlorofluorescin diacetate (DCFH-DA), propidium iodide (Calcein AM/PI), cell counting kit-8 (CCK-8), annexin V-FITC/PI, 4′,6-diamidino-2-phenylindole (DAPI), were acquired from Beyotime (Shanghai, China).

### Characterization

The morphology and structure of HPB/ATS/MB were characterized using H-7650 TEM (Hitachi, Japan) and S-4800 SEM (Hitachi, Japan). NanoBrook (Brookhaven, Germany) was used to measure the hydration particle size of HPB/ATS/MB. The powder X-ray diffraction (PXRD) patterns were recorded on a D8 ADVANCE X-ray powder diffractometer (Bruker, Germany). Thermal images were measured using a thermal imager (Hikvision, China).

### Synthesis of PB NPs

PB NPs were synthesized following the methods described in the literature [32]. In a typical procedure, K_3_[Fe(CN)_6_]·3H_2_O (131.72 mg) and PVP (3 g) were added to 40 ml of a 0.01 M HCl aqueous solution and stirred magnetically for 30 min until the solution became clear. The mixture was then reacted at 80°C for 20 h. After 24 h of aging, PBs were collected by centrifugation (13 000 rpm, 20 min) and washed three times with ethanol and deionized water. The obtained PBs were dried at 25°C for another 24 h.

### Synthesis of HPB NPs

PVP-modified hollow mesoporous Prussian blue (PVP/HPB) was synthesized using controlled chemical etching technology based on the method described in the literature. Dry PVP/PB (20 mg) and PVP (100 mg) were transferred to a 20 ml 1 M HCl solution in a Teflon vial and stirred for 4 h at room temperature. The Teflon vial was then placed in a stainless-steel autoclave and heated to 140°C for 4 h. After the reaction solution cooled to room temperature, PVP/HPB was collected by centrifugation (13 000 rpm, 20 min) and washed three times with ethanol and deionized water.

### Synthesis of HPB/ATS NPs

First, 5 mg of ATS was dissolved in 10 ml of ethanol, followed by the addition of 10 ml of HPB NPs (1 mg/ml) lyophilized powder for ultrasonic dispersion and stirring for 12 h at room temperature. Finally, HPB/ATS was collected by centrifugation (13 000 rpm, 20 min) and washed three times with ethanol and deionized water.

### Synthesis of HPB/ATS/MB NPs

About 10 mg of HPB/ATS was dissolved in 10 ml of deionized water, followed by the addition of 2 mg of MB and stirring for 24 h under dark conditions. Finally, HPB/ATS/MB NPs were collected by centrifugation (13 000 rpm, 20 min) and washed three times with ethanol and deionized water.

### Photothermal performance of HPB/ATS/MB NPs

HPB/ATS/MB solution (0, 12.5, 25, 50, 100 and 200 μg ml^−1^, 1 ml) was placed in a 1.5 ml EP tube and exposed to NIR-I (808 nm) laser (1.5 W cm^−2^, Shanghai Connect Fiber Optics Company, China). In addition, HPB/ATS/MB NPs solution (50 μg ml^−1^, 1 ml) was placed into a 1.5-ml EP tube and irradiated with an 808 nm laser at different power densities (0.50, 1.00, 1.25, 1.5, 2 W cm^−2^).

### *In vitro* cell uptake

To examine how HPB/ATS/MB enters cells, we cultured 4T1 cells on CLSM-specific culture dishes measuring 15 ml in diameter. After incubating the cells for 24 h, they underwent three PBS washes. Then, a solution of HPB/ATS/MB at 5 μg/ml was added and incubated at 37°C for 4, 8 and 12 h. After ensuring removal of residual nanoparticles and dead cells with three additional PBS washes, CLSM was used to visualize the uptake of HPB/ATS/MB into the cells.

### *In vitro* cell viability assay

Cell viability was quantified using the CCK-8 assay. In a typical experiment, 4T1 cells were cultured in 96-well plates for 24 h under suitable conditions. DMEM containing different materials was added to each well and incubated for another 24 h. Finally, after washing with a PBS solution, the medium in each well was replaced with PBS containing the CCK-8 solution and incubated for another 1–4 h. The absorbance of each well at the characteristic wavelength (450 nm) was detected with a microplate reader. To observe live and dead cells using CLSM, 4T1 cells were seeded onto CLSM dishes. The cells were observed after incubation for 24 h after adding different materials. The culture medium was removed, and live cells were stained green (*λ*_ex_ = 490 nm, *λ*_em_ = 515 nm) and dead cells were stained red (*λ*_ex_ = 535 nm, *λ*_em_ = 617 nm) using calcein solution (50 μl, 20 mM) and PI solution (50 μl, 20 mM) after washing. After 15 min of incubation, the staining solution was removed, the cells were rinsed twice with PBS and observed with CLSM.

### Biocompatibility evaluation

All animal experiments were conducted under the authorization and guidance of the Huaqiao University Animal Experiment Ethics Committee (No. A2020029). To assess the effects of the nanomedicine on normal organs, the compound was administered to mice via tail vein injection. After a 14-day treatment period, the mice were euthanized, and their heart, liver, spleen, lungs and kidneys were collected. The tissue samples were then dehydrated, cleared and embedded in paraffin to prepare tissue sections. These sections were subsequently stained with hematoxylin and eosin (H&E) and examined under a microscope for pathological changes. Additionally, blood samples were collected from the mice for hematological analysis.

### *In vivo* tumor therapy

To evaluate the therapeutic effect of HPB/ATS/MB *in vivo*, female BALB/C nude mice were randomly divided into four groups, and 4T1 cells were injected subcutaneously into the right front leg to establish a transplanted tumor model. When tumors grew to 20 mm^3^, different materials at a dose of 5 mg/kg were injected through the tail vein. After 8 h, the tumor site was irradiated with an 808 and 655 nm laser. Mouse body weight and tumor volume were measured daily after administration. All tumors were sectioned for H&E/TUNEL staining for histological analysis. The heart, liver, spleen, kidneys and lungs were also collected for H&E staining.

### Statistical analysis

Data are presented as mean ± standard deviation (*n* ≥ 3). Differences were assessed using one-way analysis of variance (ANOVA). Statistical significance was considered at *P* < 0.05 (* indicates *P* < 0.05, ** indicates *P* < 0.01, *** indicates *P* < 0.001).

## Results and discussions

**Figure 1.**
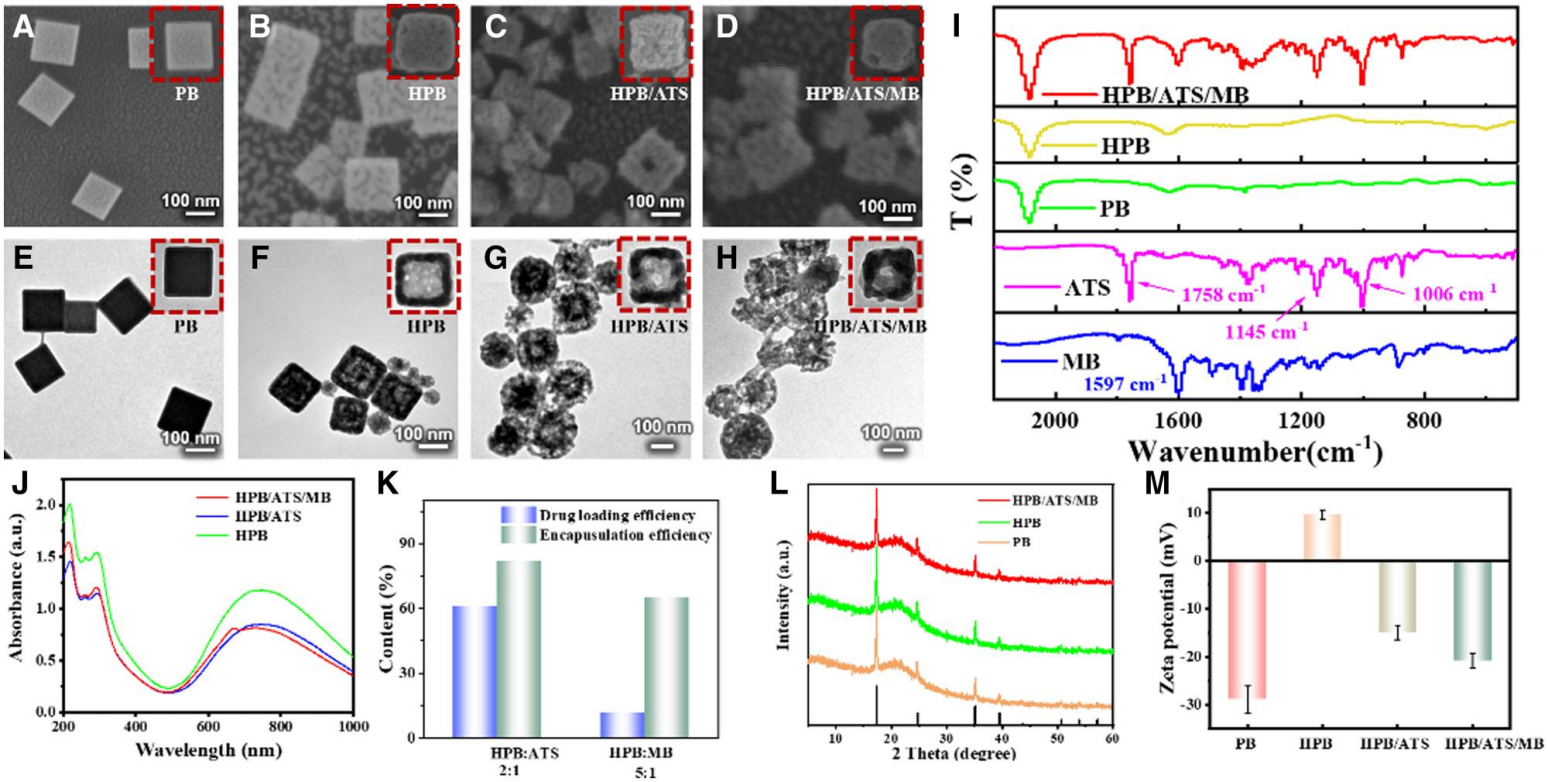
Characterization of PB, HPB, HPB/ATS and HPB/ATS/MB. (**A–D**) Scanning electron microscopy (SEM) images for PB, HPB, HPB/ATS and HPB/ATS/MB. (**E–H**) Transmission electron microscopy (TEM) images for PB, HPB, HPB/ATS and HPB/ATS/MB. (I) Fourier transform infrared (FTIR) spectra for MB, ATS, PB, HPB and HPB/ATS/MB. (**J**) Visible-near infrared (Vis-NIR) absorbance spectra for HPB, HPB/ATS and HPB/ATS/MB. (**K**) The loading efficiency and encapsulation efficiency of ATS and MB. (**L**) XRD spectra for PB, HPB and HPB/ATS/MB. (**M**) The zeta potential of PB, HPB, HPB/ATS and HPB/ATS/MB.

Characterization of PB, HPB, HPB/ATS and HPB/ATS/MB was conducted using various techniques. SEM and TEM images were obtained to examine the morphologies of the synthesized materials (Figure 1A–H). PB exhibited a solid cuboid structure with a uniform size of approximately 160 nm. HPB, obtained after etching PB with HCl, displayed a mesoporous surface and a hollow interior, facilitating the loading of drugs. SEM and TEM images of HPB/ATS and HPB/ATS/MB confirmed that the nanoparticles remained monodispersed with no significant changes in size and structure upon drug loading. Notably, the drug loading process involved agitation, ultrasound and centrifugation, resulting in a slightly irregular structure compared to HPB. However, the drug loading did not damage the nanoparticle structure or affect subsequent experiments (Supplementary Figures S1 and S2).

To confirm the successful loading of ATS and MB onto HPB, FTIR spectra of the materials and synthetic samples were measured (Figure 1I). It could be seen that PB, HPB and HPB/ATS/MB all showed a Fe^2+^-C≡N-Fe^3+^ stretching vibrational peak at 2080 cm^−1^, which is a characteristic absorption peak belonging to PB NPs [33]. This confirmed that the crystal framework of the nanoparticles was not disrupted during the etching of PB into HPB and HPB-loaded ATS and MB. Characteristic absorption peaks of MB occurring at about 1700 cm^−1^ are the stretching vibration peaks of C=S in its backbone structure, and the absorption peak at 1597 cm^−1^ is attributed to the bending vibration of the benzene ring C-C backbone in its structure [34]. The peaks of ATS at 1758 cm^−1^ are the characteristic absorption peaks of the inner peroxide bridges in its structure, and the absorption peaks at 1145 and 1006 cm^−1^ belong to the telescopic vibration peaks of ester-bonded C-O-C and ether-bonded C-O-C, respectively [35]. Additionally, characteristic absorption peaks of MB and ATS were observed in the corresponding positions of HPB/ATS/MB, confirming the successful loading of MB and ATS, respectively. The Vis-NIR absorption spectra (Figure 1J) revealed that HPB/ATS/MB exhibited an absorption peak at 650 nm, corresponding to the characteristic absorption peak of MB [36]. This provided evidence of successful MB loading. In addition, since the crystal network structure of PB is formed by the alternating arrangement of Fe^2+^ and Fe^3+^, the charge jump between the two metal ions results in a broad absorption peak in the range of 600–900 nm [37, 38] and the maximum absorption wavelength is at about 740 nm. The loading efficiency (Le) and encapsulation efficiency (Ee) of ATS and MB were determined to assess the drug-loading capacity of HPB (Figure 1K). ATS showed a Le of 60.32% and an Ee of 87.47% when the ATS-to-HPB dosage ratio was 1:2. MB exhibited a Le of 12.24% and an Ee of 65.98% at a dosage ratio of MB-to-HPB of 1:5. These results indicated that HPB had a high drug loading rate and could effectively deliver ATS and MB for photothermal and photodynamic synergistic therapy of tumors. The XRD spectra (Figure 1L) confirmed that the synthesized PB possessed a face-centered cubic structure with characteristic diffraction peaks at 17.4°, 24.5°, 35.2° and 39.5° corresponding to the crystalline surfaces of (200), (220), (400) and (420), respectively [39], consistent with the standard card JCPDS No. 01-0239. This indicated that the crystal structure of the nanoparticles remained unchanged after HCl etching and drug loading. Zeta potential measurements (Figure 1M) were conducted to assess the surface charges of PB, HPB, HPB/ATS and HPB/ATS/MB. PB exhibited a negative potential of approximately −28.9 ± 2.8 mV due to the presence of PVP coating on its surface. Upon etching PB with HCl, the potential of HPB shifted to positive (9.5 ± 1.1 mV) as the internal crystal framework exposed Fe^2+^ and Fe^3+^. Loading ATS inside HPB reversed the potential to negative (−14.9 ± 1.5 mV) due to the presence of -OH groups and $O_{2}^{-}$ in ATS. Finally, the loading of negatively charged MB resulted in a potential of −20.8 ± 1.6 mV for HPB/ATS/MB. The large absolute value of potential indicated good dispersibility, preventing aggregation and precipitation, which is advantageous for cellular uptake and the anticancer effect of the nanoparticle.

**Figure 2.**
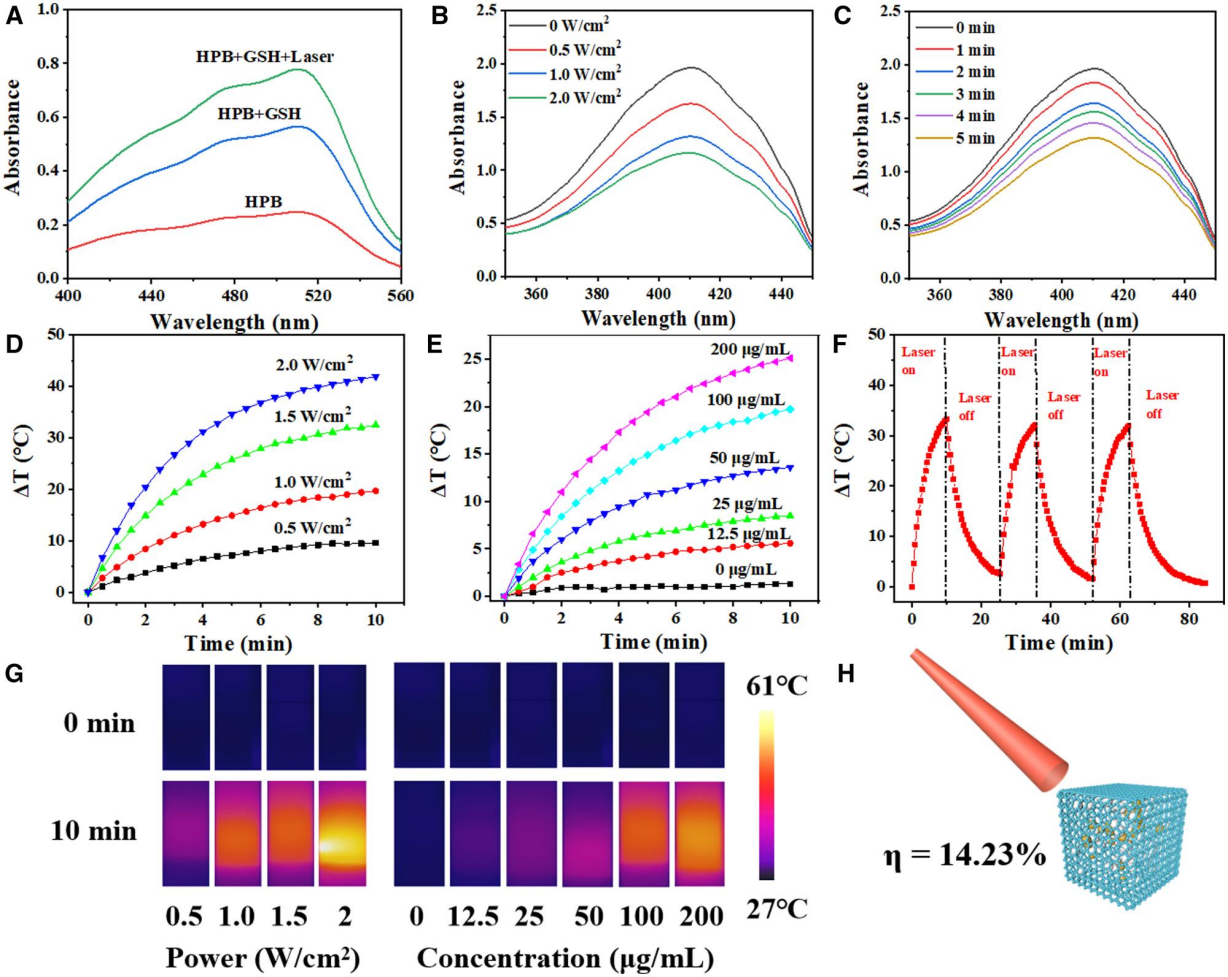
Physicochemical characterization of HPB/ATS/MB. (**A**) Comparison of ferrous ion release rates of HPB under different stimuli (GSH and laser). (**B**) Fluorescence intensity curves of DPBF and HPB/ATS/MB under varying laser power densities (0.5, 1.0, 1.5 and 2.0 W/cm^2^). (**C**) Fluorescence intensity curves for HPB/ATS/MB under different irradiation times using a 655 nm laser with a power density of 1.0 W/cm^2^. (**D**) The photothermal conversion and NIR images for a 1 ml solution of 100 μg/ml HPB/ATS/MB within a 10-min period. (**E**) The photothermal conversion for HPB/ATS/MB at different concentrations, irradiated for 10 min at a power density of 1.5 W/cm^2^. (**F**) The photothermal stability of 100 µg/ml HPB/ATS/MB. (**G**) Photothermal photos of HPB/ATS/MB at different power densities and concentrations. (**H**) The photothermal conversion efficiency of HPB/ATS/MB.

The phenanthroline compound can undergo a reaction with ferrous ions, resulting in the formation of an orange-red complex. This complex exhibits a distinct ultraviolet absorption peak at 510 nm, with the absorbance directly proportional to the release of ions. In Figure 2A, the absorbance at 510 nm for HPB solutions of the same concentration under different conditions is measured as 0.247, 0.566 and 0.779, respectively. These results indicate that the presence of photothermal and GSH leads to an increased release of Fe^2+^. GSH facilitates the reduction of Fe^3+^ to Fe^2+^, while the photothermal effect contributes to the Fe^2+^ release in HPB.

Singlet oxygen, a ROS known for inducing apoptosis and necrosis, is commonly utilized in PDT for tumor treatment. The photosensitizer MB sensitizes oxygen to generate singlet oxygen when exposed to laser irradiation. In this experiment, the generation of singlet oxygen was assessed using the DPBF fluorescence decay method. DPBF acts as an indicator of singlet oxygen, exhibiting strong absorption at 420 nm. The decrease in absorbance at 420 nm reflects the ability of HPB/ATS/MB to generate singlet oxygen upon reaction with it. As depicted in Figure 2B, the spectral curve gradually declines with an increase in power density, indicating a positive correlation between the ability of HPB/ATS/MB to generate singlet oxygen and the power density. When the power density remains constant at 1.0 W/cm^2^, the absorbance at 420 nm decreases with an increase in irradiation time (Figure 2C), suggesting that within a specific range, prolonging the irradiation time enhances the production of singlet oxygen by HPB/ATS/MB.

HPB exhibits a prominent absorption peak in the near-infrared range of 600–900 nm. Upon laser irradiation, the charge transition of Fe^2+^ and Fe^3+^ in the surface structure of HPB converts light energy into thermal energy, enabling photothermal conversion [37, 38]. To investigate the *in vitro* heating effect of HPB/ATS/MB, a 1 ml sample solution with a concentration of 100 μg/ml was subjected to varying power densities. As presented in Figure 2D, the heating effect of HPB/ATS/MB nanomaterials gradually intensifies with increasing power density. At power densities of 0.5, 1.0, 1.5 and 2.0 W/cm^2^, the temperature of HPB/ATS/MB increases by 9.6, 19.7, 32.5 and 41.9°C, respectively, after 10 min. The change in sample temperature is positively correlated with the power density of the laser. Thermal imaging photos captured by a thermal imager at the start and after 10 min (Figure 2G) visually demonstrate the temperature change. Subsequently, 1 ml of HPB/ATS/MB solutions with concentrations of 12.5, 25, 50, 100 and 200 μg/ml were examined, while ultrapure water served as the blank control, to investigate the effect of solution concentration on photothermal conversion (power density of 1.5 W/cm^2^). Figure 2E illustrates the photothermal heating curve of HPB/ATS/MB, and Figure 2G presents the thermal images of the nanoparticles before the start of laser irradiation (0 min) and at the end of irradiation (10 min). The control group (0 μg/ml) exhibits no noticeable trend in temperature change within 10 min of laser irradiation. With an increase in sample concentration, the photothermal heating effect becomes more pronounced. The temperatures of HPB/ATS/MB with concentrations of 12.5, 25, 50, 100 and 200 μg/ml are measured as 34.8, 37.9, 43.1, 49 and 53.6°C, respectively, corresponding to temperature increases of 5.6, 8.5, 13.6, 19.7 and 25.1°C, respectively. The photothermal conversion efficiency of HPB/ATS/MB is calculated to be 14.23%, indicating that it maintains a high level of photothermal conversion efficiency compared to traditional photothermal agents. Hence, the nanoparticles possess favorable photothermal properties suitable for subsequent tumor PTT. Figure 2F exhibits the temperature changes of HPB/ATS/MB after three natural cooling cycles following 10 min of laser irradiation. The figure reveals that the solution’s temperature rapidly decreases after the laser irradiation ceases, while the nanoparticles maintain excellent photothermal conversion performance during the laser switching cycle. This observation suggests that the nanoparticle structure remains unchanged after laser irradiation, demonstrating exceptional photothermal stability and efficient photothermal conversion efficiency.

To further validate the impact of carbon-based free radicals with extended half-lives, we employed DCFH-DA as a fluorescent probe to quantify the remaining free radicals within the system. The fluorescence intensity of HPB/ATS and HPB/ATS/MB, exhibited a notable enhancement in comparison to the MB group and the HPB group. Moreover, even after 24 h, a robust fluorescence signal persisted, indicating that the carbon-based free radicals generated by HPB and ATS possessed a prolonged half-life and significantly extended activity duration in comparison to hydroxyl free radicals (Supplementary Figures S3 and S4).

**Figure 3.**
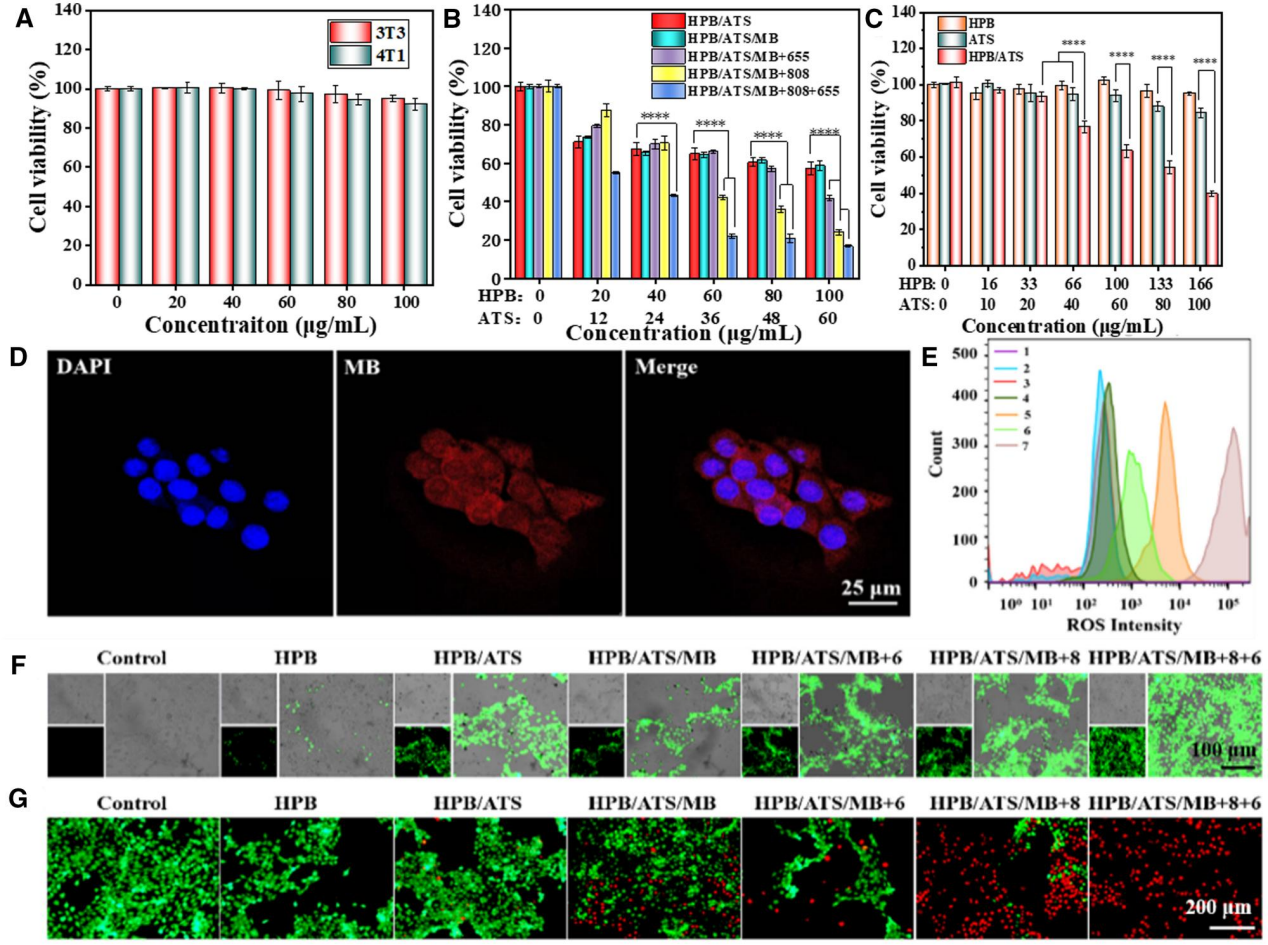
(**A**) Cell viability of 3T3 and 4T1 cells after co-culture with HPB for 24 h. (**B**) Phototherapy effects on cell viability using HPB/ATS, HPB/ATS/MB, HPB/ATS/MB + 655, HPB/ATS/MB + 808 and HPB/ATS/MB + 808 + 655 co-cultured with 4T1 cells for 24 h (****P < 0.0001). (**C**) Impact of HPB at the cellular level on the anticancer effect of ATS (****P < 0.0001). (**D**) Fluorescence localization map showing the distribution of HPB/ATS/MB in 4T1 cells after 8 h of co-culture. (**E**, **F**) ROS staining images of different experimental groups and 4T1 cells co-cultured for 8 h, along with fluorescence intensity of ROS measured by cytometry (1: Control, 2: HPB, 3: HPB/ATS, 4: HPB/ATS/MB, 5: HPB/ATS/MB + 655, 6: HPB/ATS/MB + 808, 7: HPB/ATS/MB + 808 + 655). (**G**) Live and dead staining of cells in different experimental groups and 4T1 cells co-cultured for 24 h.

To assess the biocompatibility of the synthesized HPB NPs at the cellular level, their effects on 3T3 cells and 4T1 cells were investigated. Figure 3A depicts the cell proliferation rate after co-culturing the cells with HPB NPs for 24 h. The results demonstrate a consistent impact of HPB NPs on the proliferation of both cell types. As the concentration of HPB gradually increased, there was a slight decrease in cell viability. At the maximum concentration of 100 μg/ml, the cell viability for 3T3 and 4T1 cells was 95.14 ± 1.66% and 92.12 ± 2.73%, respectively, which did not significantly differ from the control group. These findings confirm the excellent biocompatibility and lack of toxic side effects on normal cells, establishing the potential of HPB for safe cancer treatment.

Studies have revealed that introducing exogenous ferrous ions can enhance the selective toxic effects of artemisinin derivatives on cancer cells [40]. Ferrous ion release experiments have confirmed that HPB nanoparticles could successfully release ferrous ions. Next, we investigated whether the introduction of HPB could enhance the cytotoxicity of ATS at the cellular level. The cytotoxicity of HPB carrier was very low, and at a concentration of 166 μg/ml, co-culture with 4T1 for 24 h had no toxic effects on cell growth (Figure 3C). The cytotoxicity of HPB/ATS in the drug-carrying group was significantly different from that of ATS in the bare drug group when the ATS dosage was greater than 40 μg/ml, and the difference became more significant with the increase of the dosage. When the concentration of ATS was 100 μg/ml, the cell survival rates of ATS and HPB/ATS groups were 84.60 ± 2.56% and 36.69 ± 1.36%, respectively, with a difference of about 48%, indicating that the introduction of HPB could significantly enhance the killing effect of the drug ATS on tumor cells. This result confirms that iron is an activator of artemisinin derivatives in cancer cells and can mediate their anti-tumor effects. Experimental results by Xu *et al.* [41] showed that adding the iron chelator DFO significantly improved the toxicity of artemisinin while reducing the amount of intracellular ROS generated, further confirming that carbon radicals were generated due to ferrous ions and ATS, increasing ATS cytotoxicity. To evaluate the effects of 808 and 655 nm lasers on cell viability, various concentrations of nanoparticles were co-cultured with 4T1 cells for 24 h. The cell viability, determined by CCK-8 assay, was used to assess the phototherapy effect of the nano-drugs on the cells. Figure 3B shows a downward trend in cell viability with increasing drug concentration. As observed in Figure 3A, HPB alone exhibited low toxicity to cells. However, when loaded with ATS, the drug toxicity increased significantly. Co-culturing cells with 100 μg/ml HPB/ATS resulted in a 57.49 ± 3.31% decrease in cell viability after 24 h. This indicates that the introduction of exogenous Fe^2+^ activates the selective killing effect of ATS on cancer cells by generating carbon radicals [42]. The cell survival rates of the experimental groups HPB/ATS, HPB/ATS/MB and HPB/ATS/MB + 655 showed no significant differences, suggesting that the photodynamic effect relying solely on HPB-loaded MB was not sufficient. This could be due to the limited number of photosensitizer MBs loaded on the carrier. However, when the drug concentration reached 100 μg/ml, the cell survival of the HPB/ATS/MB + 655 group was reduced by approximately 16% relative to the other two groups (41.78 ± 1.35%). This increase in nanoparticles concentration enhanced the photodynamic effect.

Similarly, the cytotoxicity of the HPB/ATS/MB group significantly increased when externally stimulated by the 808 nm laser at the same concentration. At a concentration of 100 μg/ml, the cell survival rate was only 24.04 ± 1.38%. As cells produce heat shock proteins under photothermal conditions, reducing their sensitivity to high temperatures, the photothermal effect of photothermal agents is greatly diminished, necessitating alternative treatment methods for synergistic therapy [43]. The cell viability of the HPB/ATS/MB + 808 group indicated that under cellular environment and photothermal stimulation, HPB-activated ATS and the photothermal effect of HPB exerted strong effects on cells. When both the 808 and 655 nm lasers acted on HPB/ATS/MB, the cells were severely damaged due to photothermal and the massive generation of two free radicals. After 24 h, the cell viability was only 16.79 ± 0.61%. These phototherapy effects on cells demonstrate that the nano-drug-loading system developed in this project exhibits excellent cancer cell-killing ability and holds promise for anti-cancer research in animals.

DAPI, which binds to double-stranded DNA in cells and emits blue fluorescence, was used to indicate the nucleus location. The red fluorescence emitted by MB represents the location of HPB/ATS/MB. Figure 3D clearly demonstrates that the red fluorescence of the nanoparticles surrounds and aggregates around the nucleus (cytoplasm), with a significant amount falling just inside the blue nucleus. This figure illustrates that cells take up nanoparticles in large quantities after co-culturing. In addition, the red fluorescence intensity of 4T1 cells after co-incubation with nanoparticles for 8 h was significantly higher than that of 4 and 12 h (Supplementary Figures S5 and S6), indicating that most of the nanoparticles were successfully taken up by cells after 8 h of co-incubation. The phototherapy effect of nanoparticles can be fully realized with the support of 808 and 655 nm lasers, establishing a foundation for subsequent tumor treatment.

The results of the ROS detection for different experimental groups are presented in Figure 3F. No green fluorescence or dead cells were observed in the control group, indicating the absence of ROS generation. HPB/ATS produced green fluorescence in almost all cells, indicating the generation of a significant number of carbon radicals due to the introduction of exogenous Fe^2+^. Thus, compared to HPB, this group exhibited a large amount of green fluorescence. HPB/ATS/MB showed fluorescence in almost every cell in the selected area after loading the photosensitizer MB. The 655 nm laser generated singlet oxygen from MB, increasing fluorescence levels. The photothermal effect of the 808 nm laser in HPB/ATS/MB + 808 increased the production of Fe^2+^ by HPB, resulting in the generation of more carbon radicals. Therefore, compared to HPB/ATS/MB, the addition of the 808 nm laser resulted in greater ROS production in the cells. When first irradiated with the 808 nm laser and then with the 655 nm laser, carbon radicals were generated while the photosensitizer MB activated oxygen to produce singlet oxygen. Consequently, a substantial amount of green fluorescence appeared in HPB/ATS/MB + 655 + 808, almost completely covering the cells.

The fluorescence intensity of ROS was quantitatively analyzed using flow cytometry (Figure 3E). The graph represents a single-parameter analysis of ROS in cells. The x-axis represents the relative intensity of the fluorescence signal. The fluorescence intensity of HPB/ATS/MB was slightly higher than that of HPB/ATS, indicating a greater generation of ROS in cells after MB loading. Applying the 655 nm laser to HPB/ATS/MB resulted in a significant increase in ROS production compared to the 808 nm laser. This indicates that the amount of carbon radicals generated by ATS activated by exogenous Fe^2+^ under photothermal conditions is much lower than the amount of singlet oxygen generated by the photosensitizer MB. Group exhibited the highest ROS production, indicating that under the combined action of the 808 and 655 nm lasers, a large quantity of both types of ROS was generated, causing severe damage to cells. This result aligns with the cell phototherapy results, further confirming the feasibility of this research system for cancer treatment. Figure 3G illustrates the fluorescence staining results for cell viability. In the control and HPB groups, all cells showed green fluorescence, and no dead cells were observed, indicating that HPB had no toxic effect on the cells and exhibited good cytocompatibility. Some red fluorescence was observed in HPB/ATS, but it was minimal compared to the number of living cells, demonstrating mild cytotoxicity of HPB/ATS. Comparing HPB/ATS/MB with HPB/ATS, more dead cells stained with red fluorescence were observed, indicating that the double drug-loaded nanoparticles themselves have a certain cytotoxic effect. The figure for HPB/ATS/MB + 655 shows the effect of adding the 655 nm laser, where the number of dead cells was lower compared to the number of live cells. After applying the 808 nm light to HPB/ATS/MB, the number of dead cells in the visual field sharply increased, while the number of living cells significantly decreased. This shows that the photothermal effect and the generation of carbon free radicals are more effective in killing cells than the effect of adding only the 655 nm laser. When the 808 and 655 nm lasers were used together, all dead cells stained red in the visible range of the HPB/ATS/MB + 655 + 808 image, while almost no living cells were observed. This proves that the combined action of the two lasers on HPB/ATS/MB results in significant cytotoxicity, leading to a large-scale killing of tumor cells. These findings further support the potential efficacy of the nano-drug system for tumor treatment in *in vivo* experiments.

**Figure 4.**
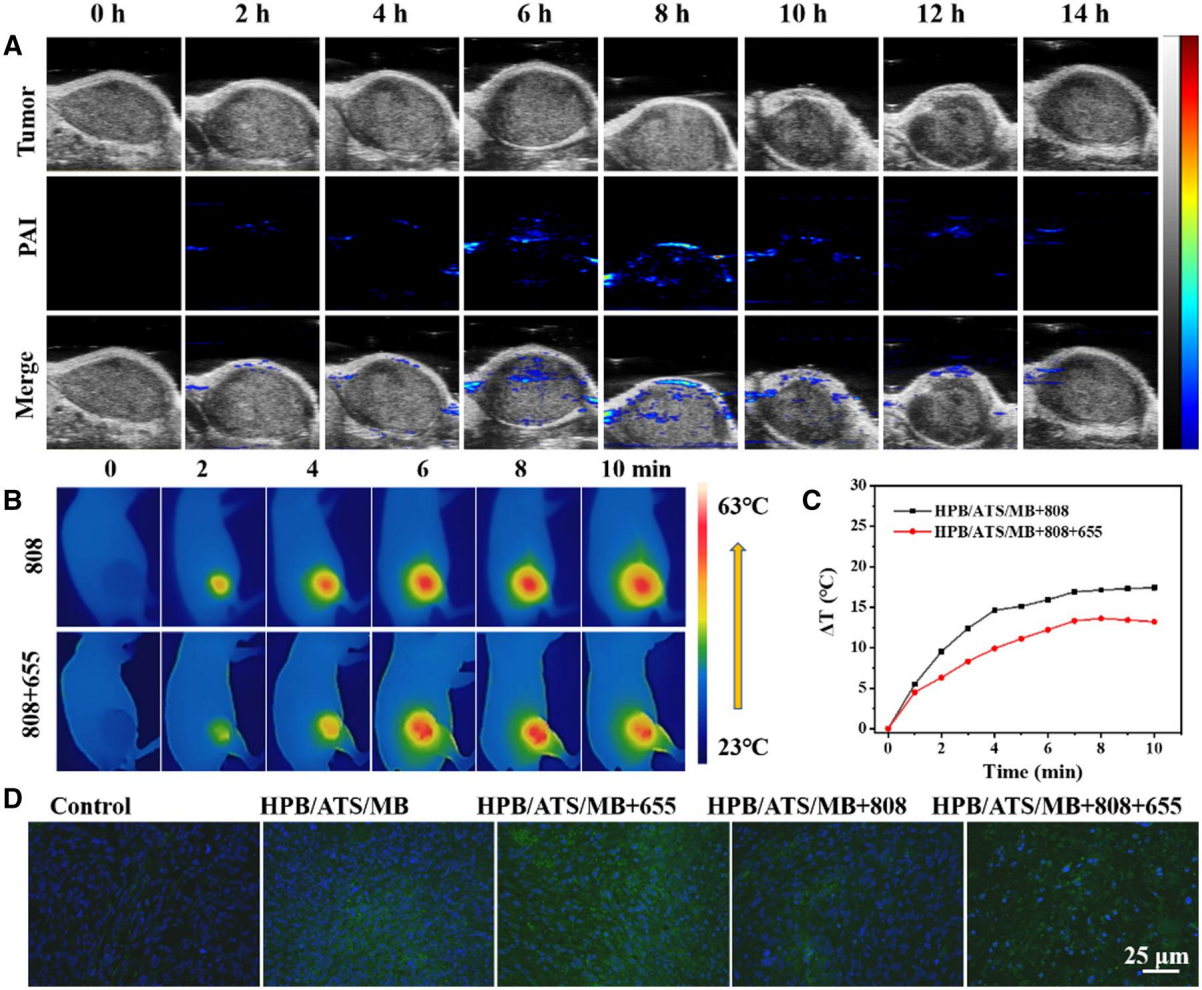
(**A**) PAI images of tumor tissue obtained 14 h after intravenous injection of HPB/ATS/MB (200 μl). (**B**) Infrared (IR) thermographic images showing 4T1 tumor-bearing mice and (**C**) *in vivo* temperature changes during a 10-min irradiation with an 808 nm laser at a power density of 1.5 W/cm^2^ in different groups. (**D**) Staining of ROS in tumor tissues from various treatment groups.

Photoacoustic imaging (PAI) of the tumor site in mice at various time points following the intravenous administration of the nanomedicine are shown in Figure 4A. Over time, the drug accumulates at the tumor site along with blood circulation, resulting in an increasingly stronger photoacoustic signal, peaking at approximately 6–8 h. Subsequently, the signal gradually diminishes, indicating drug degradation or excretion (Supplementary Figure S7). These findings demonstrate the substantial enrichment of HPB/ATS/MB at the tumor site by 8 h, suggesting that the optimal timing for subsequent phototherapy to achieve the best cancer treatment effect is 8 h after drug injection. PTT was conducted 8 h after drug injection by employing an 808 nm near-infrared laser with a laser power density of 1.5 W/cm^2^. A thermal imager recorded the temperature changes at the tumor site. Figure 4B presents a thermal image of the mouse, illustrating that the temperature of the tumor region steadily increased with prolonged irradiation time. Notably, the temperature of the tumor edge was lower than that of the tumor center, ensuring selective tumor destruction without harm to surrounding normal tissue [44]. In Figure 4B and C, the tumor temperature in the HPB/ATS/MB + 808 and HPB/ATS/MB + 808 + 655 groups rose from 37.5°C and 37.7°C to 54.9°C and 50.9°C, respectively, after 10 min of laser irradiation. This corresponded to temperature increases of 17.4°C and 13.2°C, demonstrating minimal disparity in temperature changes.

**Figure 5.**
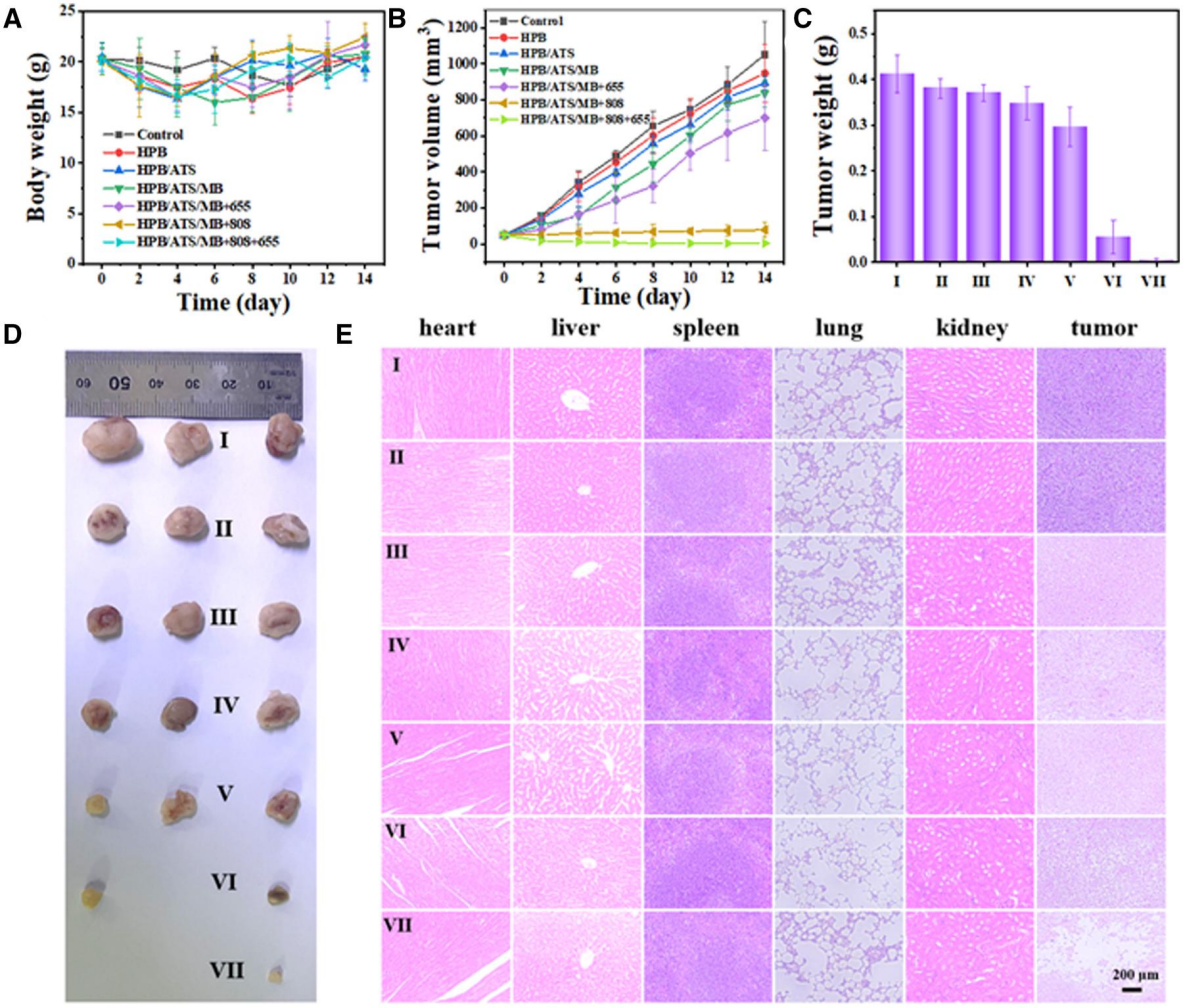
(**A**) Changes in mouse body weight over a 14-day period. (**B**) 4T1 tumor volumes after different treatment interventions. (**C**) Weights of tumors after different treatment interventions. (**D**) Representative photographs of 4T1 tumor tissues were obtained following various treatments for 14 days. (**E**) H&E staining of heart, liver, spleen, lung, kidney and tumor tissue sections after 14 days of treatment in different groups (scale bar = 200 μm). (I: Control, II: HPB, III: HPB/ATS, IV: HPB/ATS/MB, V: HPB/ATS/MB + 655, VI: HPB/ATS/MB + 808 and VII: HPB/ATS/MB + 808 + 655).

ROS staining of tumor tissues in different treatment groups is conducted to verify the ability of different nanoparticles to produce ROS in tumor sites (Figure 4D). The blue staining represents the nucleus marked by DAPI, while the green fluorescence represents ROS labeled with the H_2_-DCFDA probe. The control group exhibited minimal ROS production, whereas the HPB/ATS/MB group showed substantial green fluorescence at the interface due to the generation of carbon radicals by HPB and ATS. Upon 655 nm laser irradiation of the tumor, the MB within the nanocarriers generated a significant amount of singlet oxygen, leading to a substantial increase in ROS levels. The amount of ROS generated at the tumor site was notably lower in the HPB/ATS/MB + 808 group compared to the HPB/ATS/MB + 655 group, aligning with the cellular-level results. The HPB/ATS/MB + 808 + 655 group exhibited fewer cells, indicating the synergistic effect of photothermal and photodynamic therapies, resulting in increased ROS production (Supplementary Figure S8). These results validate the successful synthesis of a substantial amount of ROS at the tumor site using the experimentally synthesized HPB/ATS/MB nano-drug delivery system, achieving tumor eradication through the combined actions of photothermal and photodynamic therapies. The body weight of tumor-bearing mice over a 14-day period following intravenous injection were illustrated in Figure 5A. The body weight remained relatively stable within a certain range and did not exhibit significant changes. The initial mean body weights of the seven groups of mice were as follows: 20.32 ± 1.57, 20.14 ± 1.37, 20.47 ± 1.35, 20.34 ± 1.64, 20.06 ± 1.26, 20.04 ± 1.29 and 20.13 ± 1.34 g. The small differences in body weight were intentionally maintained to minimize their impact on the experimental results. After 14 days, the mice’s body weights increased to: 20.69 ± 1.84, 20.46 ± 1.02, 19.32 ± 1.17, 20.76 ± 1.34, 21.69 ± 0.99, 22.46 ± 1.39 and 20.46 ± 1.88 g, reflecting an average increase of approximately 0.37, 0.32, −1.15, 0.42, 1.63, 2.42 and 0.33 g, respectively. These observations indicate that the mice exhibited healthy growth and minimal differences in body weight. The administration of drugs and exposure to external light did not affect the mice’s food intake or overall health.

The changes in tumor volume in mice over a 14-day period are shown in Figure 5B. The tumor volume in the control group consistently increased, reaching 1052.15 mm^3^ at day 14, nearly 20 times larger than the initial volume. Furthermore, the tumors in the HPB, HPB/ATS, HPB/ATS/MB and HPB/ATS/MB + 655 groups exhibited gradual growth, albeit at a slower rate than the control group. The volume changes in these four groups followed a decreasing trend. Notably, the tumors in the HPB/ATS/MB + 808 and HPB/ATS/MB + 808 + 655 groups did not exhibit any growth during the 14-day observation period and even showed signs of regression. These findings align with the tumor-inhibitory effects observed at the cellular level, indicating that the tumor-inhibitory effect of singlet oxygen generated by the photosensitizer MB is inferior to that of long-chain carbon radicals. Figure 5C displays the tumor weight following dissection of the mice. It is evident that groups VI and VII exhibited superior tumor inhibition effects. Additionally, Figure 5D provides an optical photo of the dissected tumors, enabling a visual comparison of tumor size among the different groups of mice. H&E stained sections of the heart, liver, spleen, lung, kidney and tumor of tumor-bearing mice in each experimental group displayed intact morphology, with no significant increase in nucleus size or presence of inflammatory cells (Figure 5E and Supplementary Figure S9). These findings indicate that the synthesized nano-drug delivery system not only exhibits excellent anticancer effects but also does not induce damage to the five internal organs during cancer treatment. The system demonstrates good biocompatibility and biosafety, making it a viable option for cancer treatment in the medical field.

## Conclusion

In conclusion, we successfully synthesized PB nanoparticles with a hollow structure using the hydrothermal method. These nanoparticles were then loaded with the anticancer drug ATS and the photosensitizer MB, resulting in the development of a nano-drug delivery system named HPB/ATS/MB, which exhibits a synergistic photothermal and photodynamic effect. Within the acidic microenvironment of cells and under the influence of photothermal effects, the nanocarriers can release ferrous ions. These ions subsequently react with ATS, leading to the generation of carbon radicals with longer half-lives. Simultaneously, the photosensitizer MB generates a significant amount of ROS for PDT targeted at tumors. This study demonstrates the promising application potential of PB as a nanocarrier in the fields of ATS as an antitumor drug and photothermal/photodynamic synergistic therapy. Our research has implications for various fields, including polymer chemistry, environmental remediation, radiation shielding, high-energy-density batteries, and improved battery lifespan. These findings serve as a source of inspiration for researchers across these disciplines.

## Supplementary Material

rbae103_Supplementary_Data
