# Supplementary material for: Enhancing tumor photodynamic synergistic therapy efficacy through generation of carbon radicals by Prussian blue nanomedicine
Source: Regen Biomater. 2024 Aug 24;11:rbae103. doi: 10.1093/rb/rbae103 (PMC11434160; doi:10.1093/rb/rbae103)
Supplement: rbae103_Supplementary_Data [file rbae103_supplementary_data.docx]

***Supporting information***

**Enhancing Tumor Photodynamic Synergistic Therapy Efficacy through Generation of Carbon Radicals by Prussian Blue Nanomedicine**

Jun Zhong^1,#^, Mingzhi Zhu^1,#^ , Jiaqi Guo^1,#^, Xinyu Chen^1,#^, Ruimin Long^1^, Fabian Körte^2^, Shibin Wang^3,4,5^, Hao Chen^6,^ , Xin Xiong ^2,*^, Yuangang Liu^1,4,5,*^

^1^College of Chemical Engineering, Huaqiao University, Xiamen 361021, China

^2^NMI Natural and Medical Sciences Institute at the University of Tübingen, Markwiesenstr, 55, 72770 Reutlingen, Germany

^3^College of Materials Science and Engineering, Huaqiao University, Xiamen 361021, China

^4^Institute of Pharmaceutical Engineering, Huaqiao University, Xiamen 361021, China

^5^Fujian Provincial Key Laboratory of Biochemical Technology, Xiamen 361021, China

^6^Fujian Provincial Key Laboratory of Intelligent Identification and Control of Complex Dynamic System, Haixi Institutes, Chinese Academy of Sciences, Quanzhou 362200, China

# These authors contributed equally to this work.

E-mail: [ygliu@hqu.edu.cn](mailto:ygliu@hqu.edu.cn) (Y. Liu); [Xin.xiong@nmi.de](mailto:Xin.xiong@nmi.de) (X. Xiong);





**Figure S1.** DLS characterized hydrated particle size distribution of HPB/ATS/MB in PBS after 0 h , 24 h and 48 h.





**Figure S2.** DLS characterized hydrated particle size distribution of PB, HPB, HPB/ATS and HPB/ATS/MB in PBS.


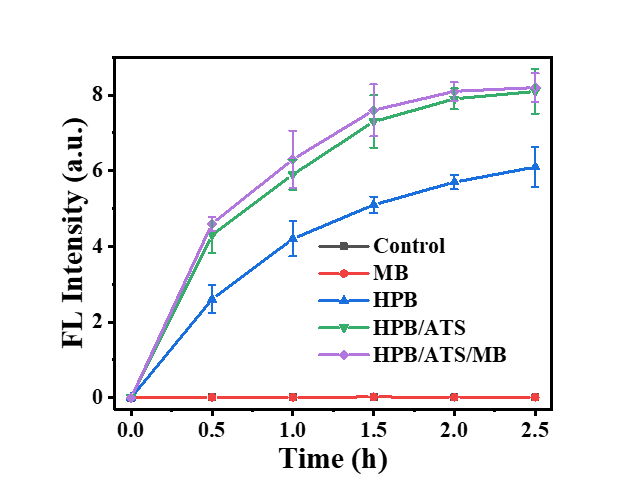


**Figure S3**. Fluorescence intensity(×10^6^) changes of DCFH at 524 nm after treatment with different materials.


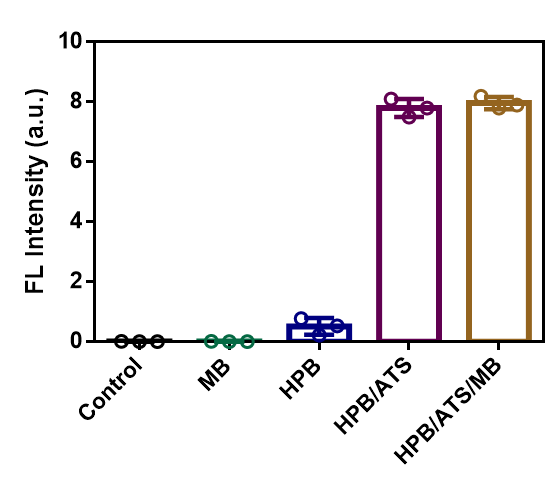


**Figure S4**. Fluorescence intensity(×10^6^) changes of DCFH at 524 nm after treatment with different materials for 24 h.


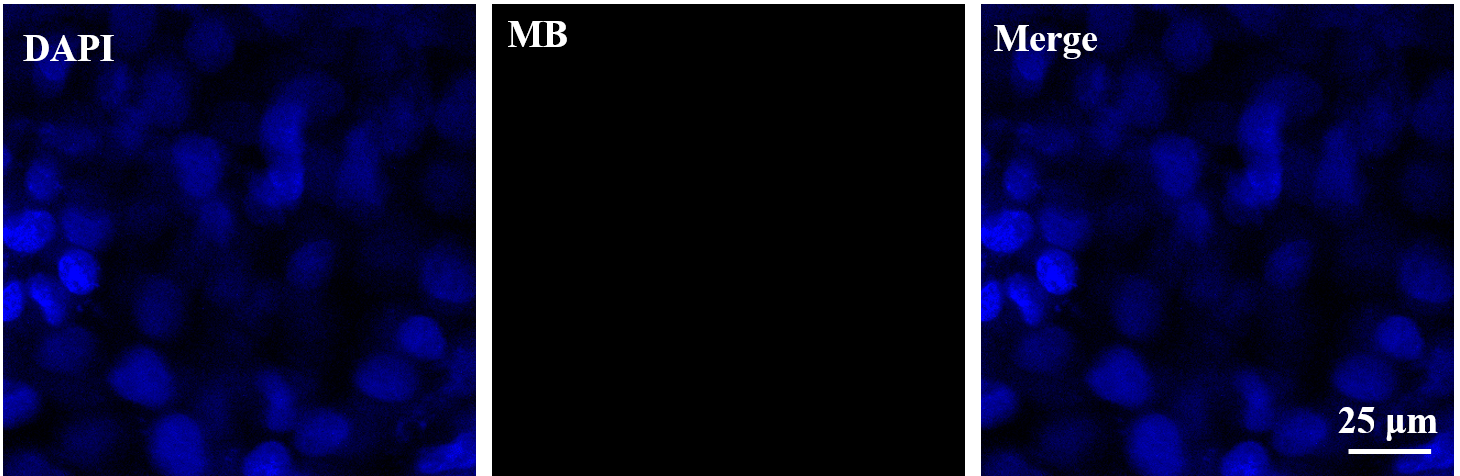


**Figure S5.** Fluorescence localization map showing the distribution of HPB/ATS/MB in 4T1 cells after 4 hours of co-culture.


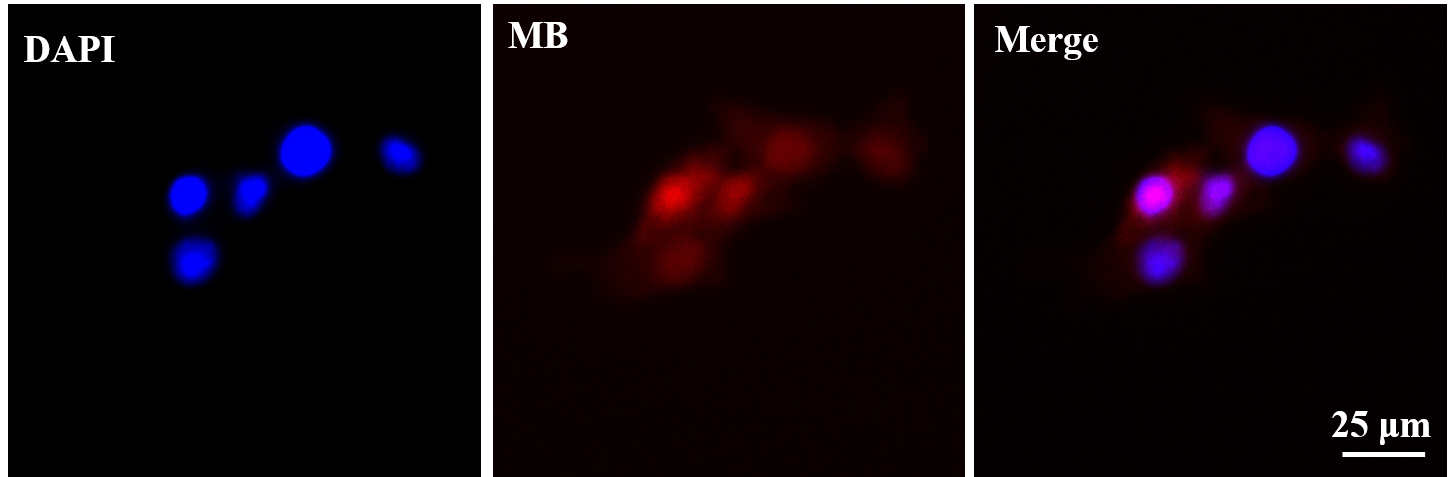


**Figure S6.** Fluorescence localization map showing the distribution of HPB/ATS/MB in 4T1 cells after 12 hours of co-culture.


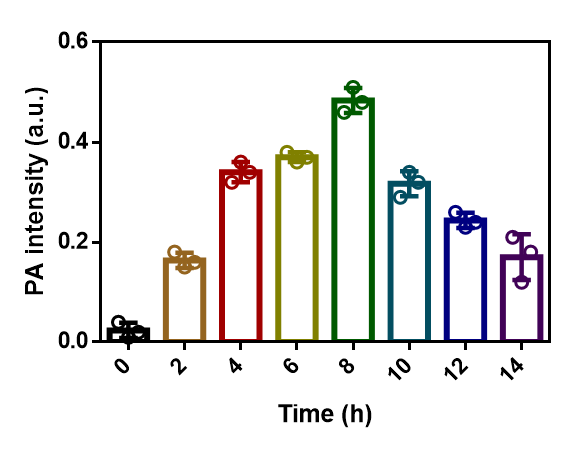


**Figure S7**. PA intensity of the tumor regions after tail-vein injection for 0, 2, 4, 6, 8, 12, and 14 h.


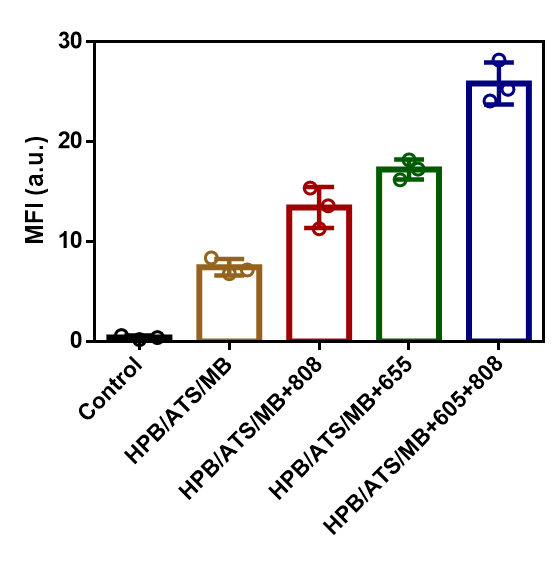


**Figure S8**. ROS fluorescence intensities of tumor sections treated with different materials.


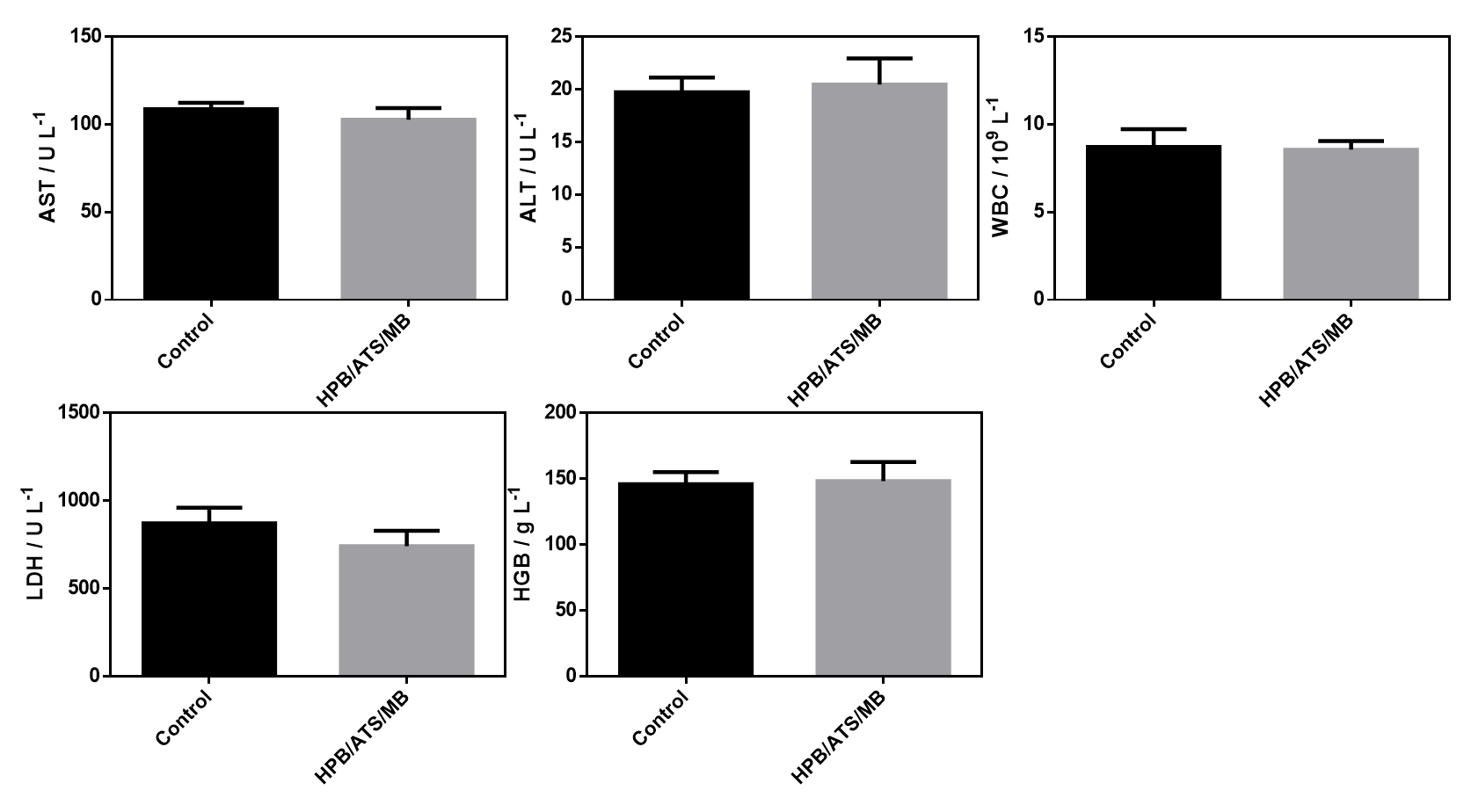


**Figure S9**. Blood biochemical index after 20 h of injections.
